# Apelin enhances the osteogenic differentiation of human bone marrow mesenchymal stem cells partly through Wnt/β-catenin signaling pathway

**DOI:** 10.1186/s13287-019-1286-x

**Published:** 2019-06-25

**Authors:** Kai Hang, Chenyi Ye, Jianxiang Xu, Erman Chen, Cong Wang, Wei Zhang, Lic Ni, Zhih Kuang, Li Ying, Deting Xue, Zhijun Pan

**Affiliations:** 1 Department of Orthopedic Surgery of the Second Affiliated Hospital, School of Medicine, Zhejiang University, No. 88, Jiefang Road, Hangzhou, 310009 China; 2 Orthopedics Research Institute of Zhejiang University, No. 88, Jiefang Road, Hangzhou, 310009 China

**Keywords:** Apelin, Osteogenesis, hBMSCs

## Abstract

**Background:**

Management of fracture healing with a large bone defect remains a tricky subject in orthopedic trauma. Enhancing osteogenesis of human bone marrow-derived mesenchymal stem cells (hBMSCs) is one of the useful therapeutic strategies for fracture healing. Previous studies have revealed that Apelin may play an important role in bone metabolism. However, its function in the osteogenesis of hBMSCs remains unclear. Therefore, in this study, we investigated the effects and mechanism of Apelin on osteogenic differentiation.

**Methods:**

We investigated the osteogenesis effects of hBMSCs by both exogenous Apelin protein and overexpression Apelin in vitro. Cell proliferation assay was used to assess the effect of Apelin on the proliferation of hBMSCs. ALP staining and Alizarin Red staining were used to evaluate ALP activity and mineral deposition respectively. qPCR and Western blotting analysis were used to detect the expression of target genes and proteins. In vivo, a rat tibial osteotomy model was established; radiographic analysis and histological evaluation were used to confirm the therapeutic effects of Apelin in fracture healing. Statistical significance was determined by two-tailed Student’s *t* test when 2 groups were compared. When more than 2 groups were compared, one-way ANOVA followed by Bonferroni’s post-hoc test was used. And two-way ANOVA, followed by Bonferroni multiple comparisons post-hoc test, was performed when the treatment groups at different time points were compared.

**Results:**

The addition of exogenous Apelin protein or overexpression of Apelin promoted osteoblast differentiation of hBMSCs in vitro. Increased mineral deposits were observed after treatment with extracellular Apelin protein or after the upregulation of Apelin. Moreover, β-catenin levels were upregulated by Apelin. The enhancement of osteogenic differentiation induced by Apelin was attenuated by specific Wnt/β-catenin signaling pathway inhibitors. In a rat tibial osteotomy model, local injection of exogenous Apelin protein improved bone healing, as demonstrated by imaging and histological analyses.

**Conclusions:**

Taken together, these findings indicate that Apelin regulates osteogenic differentiation of hMSCs partly via the Wnt/β-catenin signaling pathway and effectively promotes fracture healing.

**Electronic supplementary material:**

The online version of this article (10.1186/s13287-019-1286-x) contains supplementary material, which is available to authorized users.

## Introduction

Bone marrow-derived mesenchymal stem cells (BMSCs) are multipotent stromal cells that possess self-renewal capabilities and are able to differentiate into a variety of cell types, including osteoblasts, chondrocytes, adipocytes, and myocytes [1, 2]. Furthermore, BMSCs can be further induced to form tissues of mesodermal origin, including bones, cartilages, ligaments, muscles, tendons, and neurons [3]. The degree to which the culture will differentiate is determined by how the differentiation is induced, and varies among individuals [4]. Owing to these properties, BMSCs have been increasingly applied in regeneration medicine alone or in the form of a complex [5, 6].

The number of orthopedic trauma patients has been increasing along with the development of modern industries, and the treatment of patients with impaired fracture healing remains one of the most challenging clinical problems in trauma management [7]. There are two major fracture healing pathways—primary healing (also known as direct healing) and secondary healing (also known as indirect fracture healing). Secondary healing is the most common form of bone healing and consists of three major phases: (1) reactive phase, (2) reparative phase, and (3) remodeling phase [8]. After a fracture, acute inflammatory reactions release multiple initial inflammatory cytokines that dramatically enhance mesenchymal cell recruitment and osteogenesis [9, 10].

Apelin is the endogenous ligand for APJ, which is a seven trans-membrane G protein-coupled receptor [11] widely distributed in the limbs, heart, brain, adipose tissue, and kidney [12–14]. The Apelin/APJ system performs a broad range of activities in multiple organ systems. Apelin consists of numerous isoforms including Apelin-12, Apelin-13, Apelin-17, and Apelin-36, which are all derived from the C-terminal fragment of the Apelin pre-proprotein with 77 amino acids [15]. Among all these isoforms, Apelin-13 and its pyroglutamate-modified form Pyr-Apelin-13 are the most active isoforms that bind to the APJ receptor [16]. Apelin-13 exhibits protective effects in ischemic heart diseases, as well as anticonvulsive and neuroprotective properties. Meanwhile, Apelin-13 participates in the regulation of glucose homeostasis [17, 18]. Previous studies have demonstrated that Apelin-13 stimulates human osteoblast proliferation and suppresses serum deprivation-induced apoptosis of MC3T3-E1 cells [19, 20].

In this study, we investigated the effects of exogenous Apelin-13 on osteogenesis differentiation of human bone marrow-derived mesenchymal stem cells (hBMSCs). We found that Apelin-13 enhanced osteogenic differentiation of hBMSCs through the Wnt/β-catenin signaling pathway in vitro. We studied a rat tibial defect model treated with exogenous Apelin-13 by local injection and observed that Apelin-13 promoted healing of the bone defects in vivo.

## Materials and methods

### Cell culture and reagents

hBMSCs which can differentiate into osteoblasts, chondrocytes, and adipocytes under specific inductive conditions were purchased from Cyagen Biosciences (Guangzhou, China). Adherent hBMSCs were cultured in culture flasks with hMSC growth medium (Cyagen Biosciences, Guangzhou, China) at 37 °C with 5% CO_2_ in an incubator. hBMSCs were passaged when reached at 80–90% confluence. Cells from passages 3–8 were used in subsequent experiments. Recombinant human Apelin-13(rhApelin-13) (Abcam, MA, USA) and recombinant DKK-1 (R&D Systems) were purchased from Sino Biological. We used a DKK1 of 0.5 μg/ml based on a previous study [21].

### Cell proliferation assay

To assess the effect of recombinant Apelin-13 and APELIN overexpression on the proliferation of hBMSCs, related cells were seeded into 96-well plate (5000/well). After 24 h adhering, the medium was changed by 10% Cell Counting Kit-8 (CCK-8, Dojindo, Kumamoto, Japan) in 100 μl low-sugar Dulbecco’s modified Eagle’s medium (L-DMEM) for 4 h at 37 °C. Absorbance at 450 nm, which is proportional to cell proliferation, was measured by a microplate reader (ELX808; BioTek, USA).

### Osteogenic differentiation protocol

hBMSCs were cultured in growth medium (L-DMEM; 10% FBS (100-125, Gemini, USA) and 100 IU/ml penicillin/streptomycin) in 6- or 12-well cell culture plates at a density of 3 × 10^4^/cm^2^ and incubated for 72 h at 37 °C with 5% CO_2_. The cells were then cultured in osteogenic induction medium (L-DMEM with 10% FBS, 100 nM dexamethasone, 100 IU/ml penicillin/streptomycin, 10 mM β-glycerophosphate, and 0.2 mM ascorbic acid). The cells were maintained by changing fresh osteogenic induction medium every 2–3 days.

### ALP staining and ALP activity assay

Cells were cultured in 12-well plates with osteogenic induction medium for 3 or 5 days. For ALP staining, cells were fixed with 4% paraformaldehyde for 20–30 min. Cells were then washed by double distilled water (ddH_2_O) three times and stained by Alkaline Phosphatase Color Development Kit (Beyotime, Shanghai, China). To measure ALP activity, cells were lysed with lysis buffer consisted of 20 mM Tris-HCl (pH 7.5), 1% Triton X-100, and 150 mM NaCl. ALP activity was determined by ALP activity assay (Beyotime). Finally, the conversion color of p-nitrophenyl phosphate was measured after 3 and 5 days of culture in an osteogenic medium at 405/650 nm.

### Alizarin Red staining

Mineral deposition was assessed by Alizarin Red staining (ARS) (Cyagen Biosciences) after the induction of osteogenic differentiation. Cells were fixed with 4% paraformaldehyde for 20–30 min at room temperature and subsequently washed with ddH_2_O three times. The cells were incubated with a 0.2% solution of Alizarin Red for 5–10 min at room temperature, then rinsed with ddH_2_O. The stain is incubated with 10% cetylpyridinium chloride (Sigma, Shanghai, China) for 1 h and collected the solutions, then 200 μl was plated on a 96-well plate and then was read at 560 nm by a microplate reader (ELX808; BioTek). The results were normalized to total protein concentration.

### RNA isolation and qPCR

Total cellular RNA was isolated with RNAiso reagent (Takara, Dalian, China), and the solution was quantified by measuring the absorbance at 260 nm (NanoDrop 2000; Thermo Fisher Scientific). According to the manufacturer’s instructions of the PrimeScript RT Master Mix (Takara), first-strand cDNA was synthesized. Total RNA (≤ 1000 ng) was reverse-transcribed into cDNA using the Double-Strand cDNA Synthesis Kit (Takara) in a reaction volume of 20 μl. One microliter cDNA was used for qPCR. All gene transcripts were quantified by qPCR by SYBR Green PCR Master Mix (Takara) on ABI StepOnePlus System (Applied Biosystems, Warrington, UK). mRNAs of target genes and the housekeeping gene (GAPDH OR 18S) were quantified in separate tubes. All primers were synthesized by GENEray (Shanghai, China). The cycle conditions were as follows: 95 °C for 30 s then 95 °C for 5 s for 40 cycles and 60 °C for 30 s. The relative target gene expression levels were calculated using the 2^−ΔΔCt^ method.

### Western blotting analysis

Cells were lysed in RIPA buffer combined with proteasome inhibitor and phosphatase inhibitors (Beyotime). Equal amounts of proteins were separated by 10% or 12% sodium dodecyl sulfate polyacrylamide gel electrophoresis, then separated target proteins were transferred to a polyvinylidene fluoride membrane (Millipore, Shanghai, China). After blocking in 5% non-fat milk for 60 min, the membranes were incubated overnight at 4 °C with antibodies specific to GAPDH (1:2000; Abcam, Shanghai, China), APELIN (1:2000; Abcam, Shanghai, China), RUNX2 (1:1600; Abcam, Shanghai, China), COL1A1 (1:1000; Abcam, Shanghai, China), non-phosphorylated (active) β-catenin (1:1000; Abcam, Shanghai, China), or total β-catenin (1:1000; Abcam, Shanghai, China). After washing with TBST three times (10 min each), the membranes were incubated with secondary antibodies (horseradish peroxidase-conjugated, Beyotime) for 1 h at room temperature. After washing three times with TBST, proteins were detected using enhanced chemiluminescence blotting reagents (Millipore). Signal intensity was measured by Bio-Rad XRS chemiluminescence detection system (Bio-Rad, Hercules, CA, USA).

### Lentiviral packaging and cell infection

Lentiviral overexpression APELIN (APELIN overexpression group (OE)) particles and lentiviral GFP particles, used as the control group (APELIN overexpression control group (OE-NC)), were prepared by Cyagen Biosciences (Guangzhou, China). Forty to 50% confluent hBMSCs were incubated with lentiviral particles and 3 μg/ml polybrene in growth medium at a multiplicity of infection of 50 which is used as the optimized amount of virus determined by the GFP expression after lentiviral GFP particles infection. After 12 h, the culture medium was changed. Three days later, transfected cells were passaged for use in subsequent experiments. The expression of APELIN was verified by qPCR and Western blotting analyses.

### Immunofluorescence analysis

Cells were cultured in a 12-well plate with induction medium and evaluated for RUNX2 and APELIN using a fluorescence microscope (EU5888; Leica, Germany) as follows. Cells were fixed in 4% paraformaldehyde for 20 min at room temperature, permeabilized for 30 min in 0.2% Triton X-100, and blocked for 30 min in 2% bovine serum albumin. Fixed cells were washed and incubated overnight with anti-RUNX2 (1:1600; Abcam, Shanghai, China), APELIN (1:2000; Abcam, Shanghai, China), or COL1A1 (1:1000; Abcam, Shanghai, China). After washing three times with ddH2O, cells were incubated with a fluorescence-conjugated secondary antibody (Beyotime) for 1 h, the nuclei were stained with 4′,6-diamidino-2-phenylindole (KeyGen Biotech, Nanjing, China) for 5 min. Target proteins were observed under a fluorescence microscope (Leica).

### In vivo evaluation in animals

All Sprague-Dawley (SD) rats were purchased from the Academy of Zhejiang Province Medical Sciences. All animal experiments were in accordance with the Animal Care and Use Committee guidelines of Zhejiang University. All experimental procedures were in accordance with the Institutional Animal Care Use Committee at the second affiliated hospital of Zhejiang University (approve number: 2018-078).

In total, 18 male SD rats (8-week-old, approximately 200 g) were used to establish rat tibial defect models. All rats were divided randomly and evenly into three groups: blank group, PBS (negative control group treated with PBS) group, and Apelin group (*n* = 6 per group). All surgical procedures were performed by two experienced orthopedic surgeons. The tibial defect model was established as reported previously [22, 23]. Rats were anesthetized intraperitoneally with 0.3% pentobarbital sodium (Sigma) at a dose of 30 mg/kg body weight. After anesthesia, an incision was made lateral to the tibia, away from the bone. A 1.3-mm intramedullary fixation pin was inserted inside the medullary canal of the tibia for fixation. A 1.5-mm-diameter tibial defect was made in all SD rats approximately 7 mm from the proximal tibial growth plate by a hollow drill and punched through the cortex of the bone. The same leg was used for each group. Then rhApelin-13(50 μg) was injected locally at the fracture site on days 0, 3, 5, 8, and 11 (i.e., immediately, 72 h); PBS was used as a vehicle control. Animals were killed 6 weeks after surgery and collected the samples; then, samples were fixed in 4% paraformaldehyde for 72 h at room temperature for use in subsequent experiments.

### Radiographic analysis

For the microcomputed tomography (μCT) evaluation, tibia samples were scanned by a μCT-100 imaging system (Scanco Medical, Brüttisellen, Switzerland) with X-ray energy settings of 70 kVp, 1024 reconstruction matrix, and 14.8 μm slice thickness with an exposure time of 300 ms. The Tb. N and BV/TV were evaluated by three-dimensional standard microstructural analysis [24].

### Histological evaluation

After μCT scanning, specimens (*n* = 3 for each group) were decalcified with 10% ethylene diaminetetra acetic acid (EDTA, Sigma) with 0.1 M PBS for at least 2 months, with a solution change once a week. Thereafter, the specimens were embedded in paraffin in accordance with the standard procedures. Serial sections (4 μm thick) were cut and mounted onto polylysine-coated glass slides, deparaffinized, and then stained with HE, and Masson’s trichrome was performed separately on consecutive tissue sections in accordance with our previous studies [25]. Finally, images were obtained on a traditional light microscopy (Leica DM4000B; Leica, Solms, Germany).

### Statistical analysis

Statistical analysis was performed with SPSS 19.0 software (IBM, Armonk, NY, USA). All experiments were performed at least in triplicate, and the data are presented as means ± SD. Statistical significance was determined by two-tailed Student’s *t* test when 2 groups were compared. When more than 2 groups were compared, one-way ANOVA followed by Bonferroni’s post-hoc test was used. And two-way ANOVA, followed by Bonferroni multiple comparisons post-hoc test, was performed when the treatment groups at different time points were compared. A value of *P* ≤ 0.05 was considered significant.

## Results

### Endogenous Apelin expression and the influence of exogenous recombinant Apelin-13 on proliferation and osteogenesis differentiation of hBMSCs

**Fig. 1:**
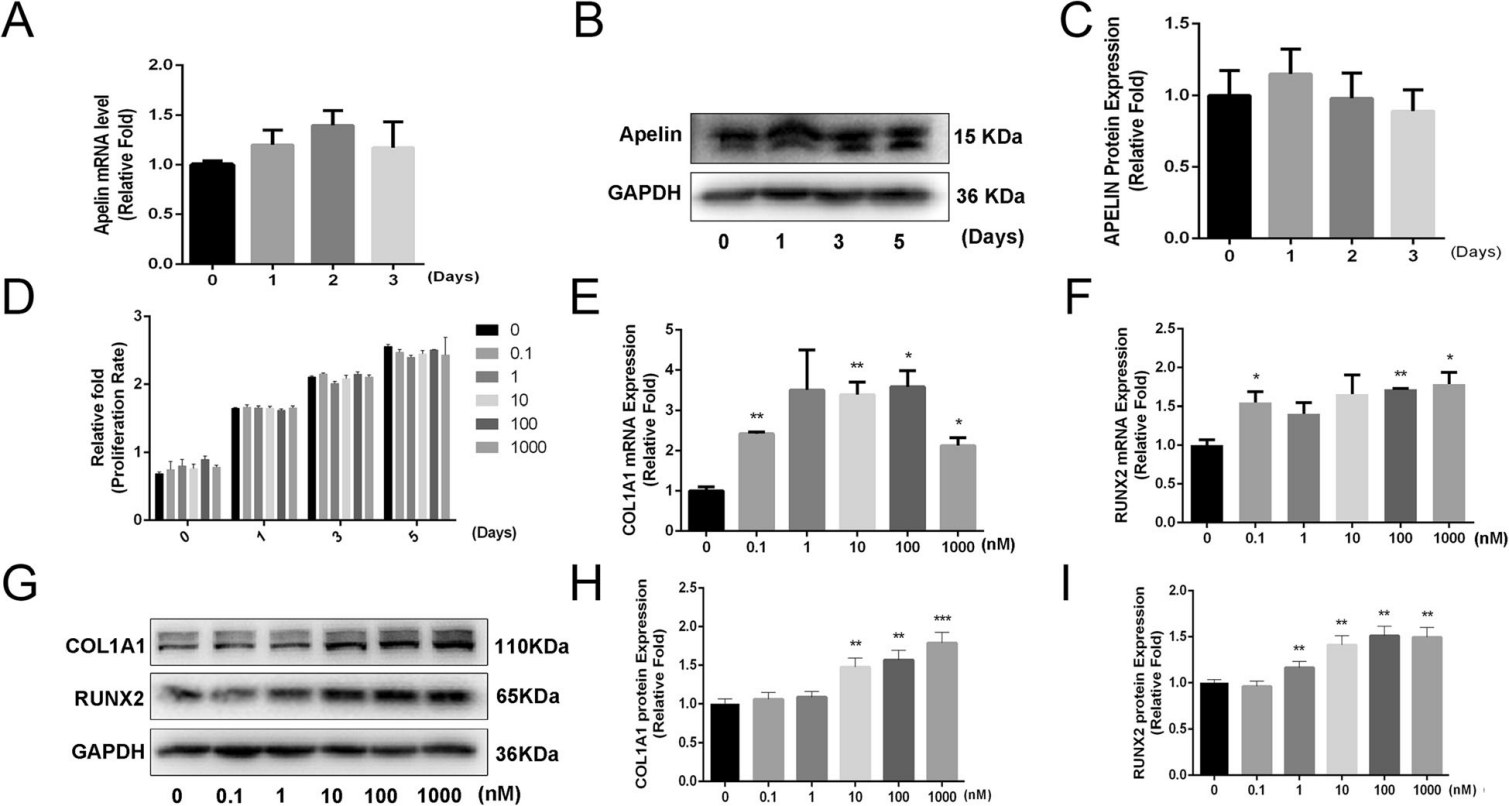
Endogenous Apelin expression and the influence of exogenous recombinant Apelin-13 on the proliferation and osteogenesis differentiation of hBMSCs. **a**–**c** Apelin expression level remained constant during osteogenic differentiation of hBMSCs. **d** hBMSC proliferation was examined by the CCK-8 assay. **e**–**i** hBMSCs were incubated with different concentrations (0–1000 nM) of Apelin-13 for 3 days. The expression of RUNX2 and COL1A1 were significantly upregulated by Apelin-13. All data are expressed as mean ± SD. Assays were performed in triplicate. *P < 0.05 and **P < 0.01 compared with the control group

To determine the expression level of Apelin associated with osteogenic differentiation of hBMSCs, we compared endogenous Apelin expression between undifferentiated and differentiated hBMSCs. In comparison with undifferentiated hBMSCs, mRNA and protein levels of Apelin showed no significant change after osteogenic differentiation on days 0, 1, 3, and 5 (Fig. 1a–c). To determine whether exogenous Apelin-13 influences the proliferation of hBMSCs, we performed the CCK-8 assay after the addition of different concentrations of Apelin-13 (0–1000 nM) during days 0, 1, 3, and 5. No significant difference was detected in cell number among different concentrations of Apelin-13 (Fig. 1d). Then, we examined the effects of Apelin-13 on the differentiation of hBMSCs and found the expression level of specific osteogenesis-related genes, including collagen type I alpha 1 (COL1A1) and runt-related transcription factor 2 (RUNX2), increased dramatically after treatment with at least 0.1 nM Apelin-13 on day 2. No obvious difference was observed after treatment with 1000 nM Apelin-13 in comparison with 100 nM Apelin-13 (Fig. 1e, f). COL1A1 and RUNX2 protein levels also increased significantly with at least 1 nM Apelin-13 on day 2 (Fig. 1g–i). Afterwards, we used a concentration range of 0 to 100 nM to study the effects of Apelin-13 on osteogenesis differentiation of hBMSCs.

### Apelin-13 increased the expression levels of osteo-specific genes and proteins and enhanced calcium deposit formation

To assess the roles of Apelin-13 in osteogenic differentiation, we determined the expression levels of osteo-specific genes and proteins including alkaline phosphatase (ALP), collagen type I alpha 1 chain (COL1A1), runt-related transcription factor 2 (RUNX2), and osteocalcin (OCN) by Western blot and quantitative real-time PCR (qPCR) analyses.

**Fig. 2:**
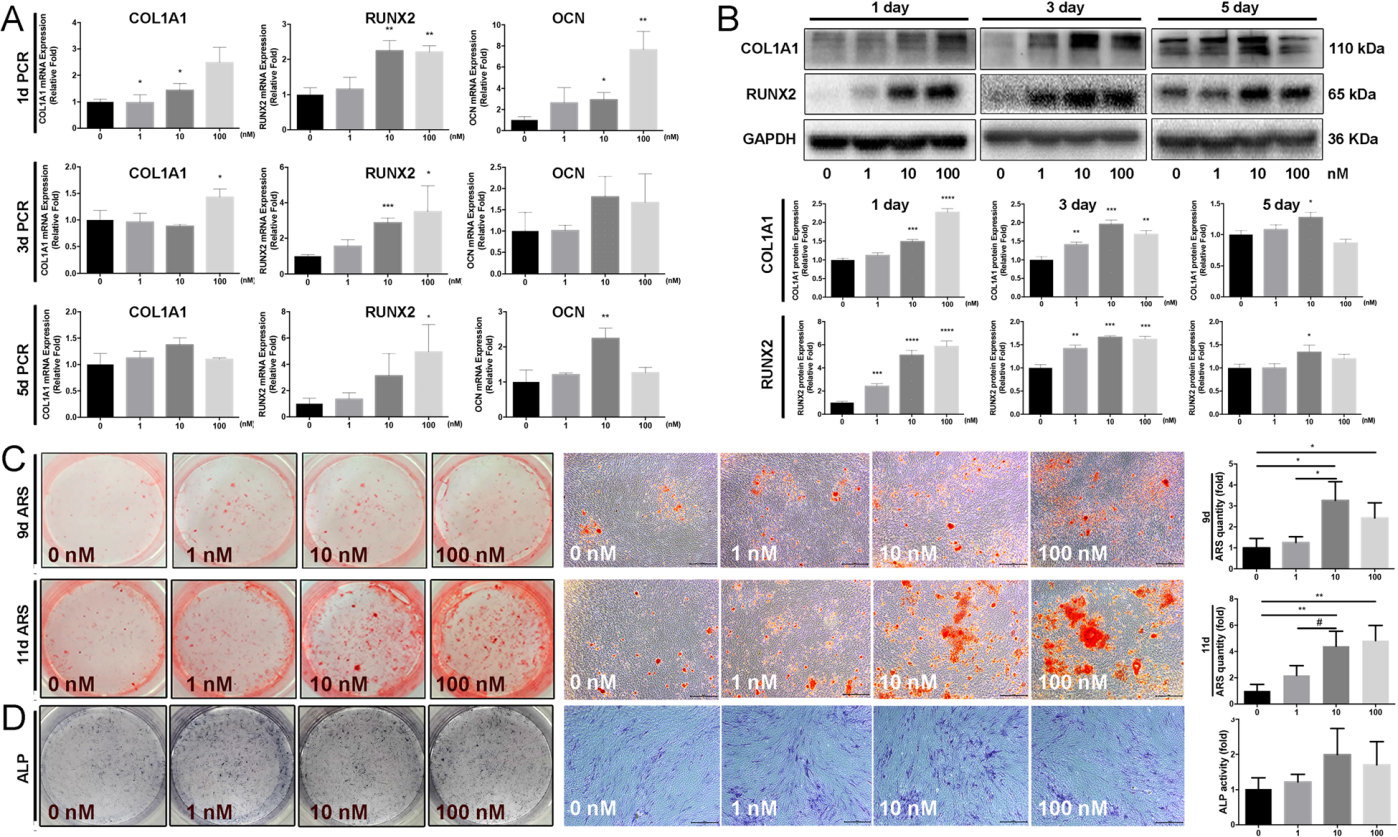
Effects of recombinant Apelin-13 on osteogenic differentiation of hBMSCs. **a** Relative mRNA expression of osteo-specific genes (COL1A1, RUNX2, and OCN) on days 1, 3, and 5 of osteogenesis. The mRNA expression levels were normalized to that of 18S ribosomal RNA. **b** Relative expression of osteo-specific proteins (RUNX2 and COL1A1) on days 1, 3, and 5 of osteogenesis. Protein expression levels were normalized to that of GAPDH. **c** Apelin-13 promoted hBMSC mineralization. The cells were incubated with different concentrations of Apelin-13 for 9 and 11 days. Calcium deposits were identified by Alizarin Red S staining. Compared with the control group, Apelin-13 enhanced mineralization dramatically. Scale bar, 500 μm. Alizarin Red S-stained area was determined by measuring the absorbance at 560 nm. **d** ALP staining on day 3 of osteogenesis and ALP activity detection on day 3 of osteogenic differentiation. Scale bars, 500 μm. Data are expressed as mean ± SD. Assays were performed in triplicates. *P < 0.05, ^#^*P* < 0.05, **P < 0.01, and ***P < 0.001 compared with the control group

Compared with the control group, Apelin-13 (ranging from 0 to 100 nM) significantly promoted the mRNA expression of COL1A1, RUNX2, and OCN during osteogenesis differentiation at days 1, 3, and 5 (Fig. 2a), whereas the mRNA expression of ALP markedly increased at days 1 and 3 (Additional file 1: Figure S2A). Furthermore, Western blot revealed that the protein levels of COL1A1 and RUNX2 were also remarkably higher following the increased mRNA expression at days 1, 3, and 5 (Fig. 2b).

We examined the calcium deposits that formed during late-stage osteogenic differentiation and maturation of osteoblasts by ARS. On days 9 and 11, Apelin-13 (0–100 nM) was found to considerably increase calcium deposits compared with the control group (Fig. 2c). Meanwhile, treatment with Apelin-13 during osteogenesis resulted in no significant difference of the ALP activity (Fig. 2d). Immunofluorescence (IF) assay revealed a significant increase in COL1A1 and RUNX2 protein expression after treatment with Apelin-13 (Additional file 2: Figure S2B).

### Apelin-13 promotes osteogenic differentiation of hBMSCs partly via the Wnt/β-catenin signaling pathway

**Fig. 3:**
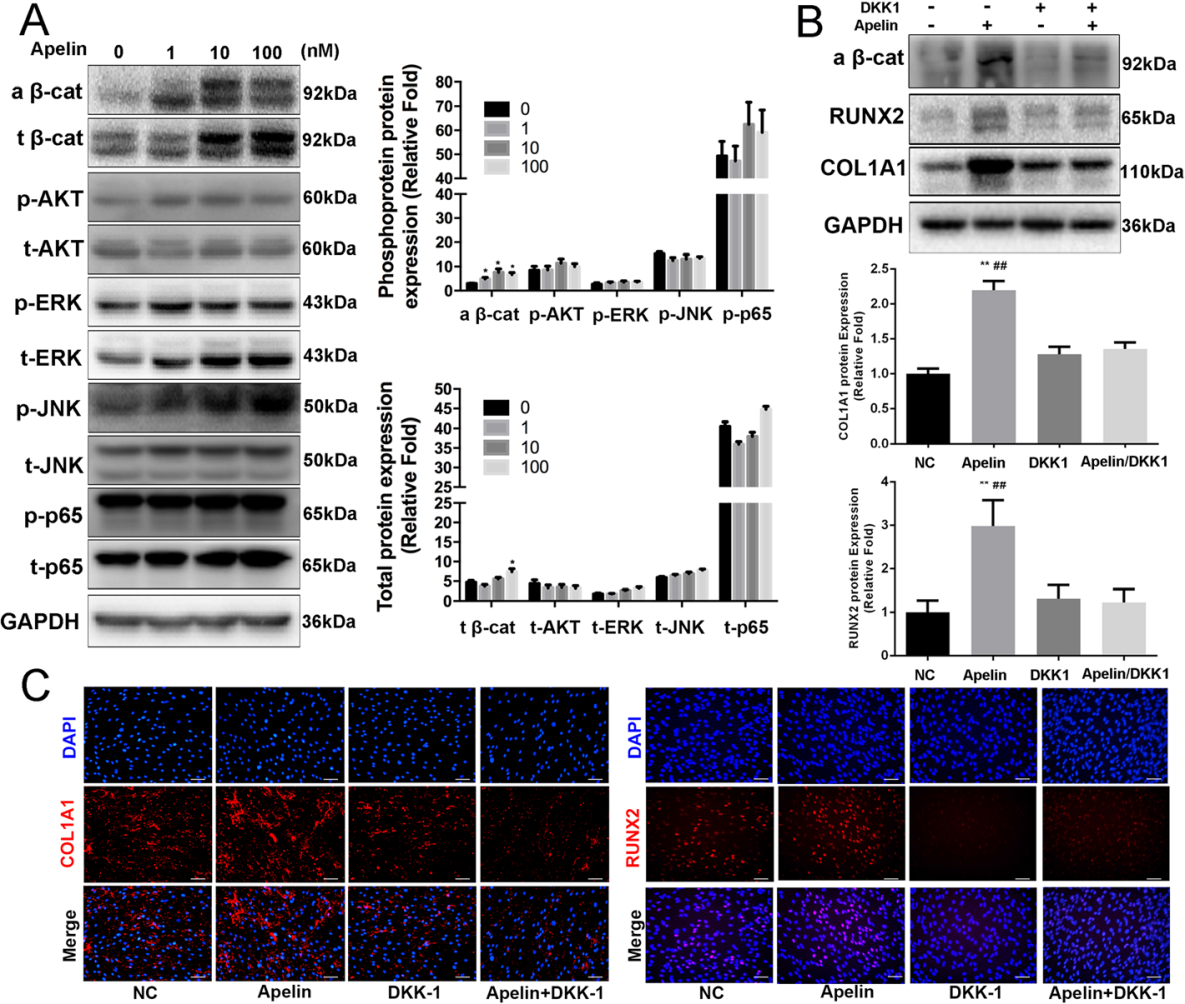
Apelin-13 activated the Wnt/β-catenin signaling pathway. **a** Comparison of signaling pathway-related protein levels by Western blot analyses. hBMSCs were incubated with different concentrations of Apelin-13 during osteogenic differentiation on day 3. Protein expression levels were normalized to that of GAPDH. **b** Increased expression of osteo-specific proteins (COL1A1 and RUNX2) due to the inhibition of exogenous recombinant Apelin-13 by DKK1. Protein expression levels were normalized to that of GAPDH. **c** Immunofluorescence staining for RUNX2 and COL1A1. Scale bars, 100 μm. Data are expressed as mean ± SD. Assays were performed in triplicate. *P < 0.05, **P < 0.01, and ^##^*P* < 0.01, compared with the control group

To elucidate the specific signaling pathways through which Apelin-13 regulates hBMSC osteogenic differentiation, we used Western blot to examine the common signaling pathways involved in osteogenesis, including the PI3K/AKT signaling pathway, the MAPK signaling pathway, and the Wnt/β-catenin pathway. The levels of total β-catenin and active β-catenin both increased after Apelin-13 (0–100 nM) treatment on day 3 of osteogenic differentiation. No significant changes were observed for the MAPK signaling pathway or the PI3K/AKT signaling pathway (Fig. 3a).

To further verify the involvement of Wnt/β-catenin signaling pathway in the regulation of hBMSC osteogenic differentiation by Apelin-13, the Apelin-13-induced inhibitory effects of this pathway on osteogenesis were analyzed. After treatment with an appropriate concentration (500 ng/ml) of DKK1, an effective inhibitor of the Wnt/β-catenin signaling pathway, we found almost complete abrogation of the promotive effect on RUNX2 and COL1A1 expression induced by Apelin-13(100 nM) on day 3 (Fig. 3b).

Moreover, immunofluorescence assay revealed that the increases in RUNX2 and COL1A1 protein expression by Apelin-13(100 nM) were suppressed by DKK1 (500 ng/ml) on day 3 (Fig. 3c).

### Endogenous Apelin overexpression increased the levels of osteo-specific proteins, enhanced ALP activity, and calcium deposit formation

**Fig. 4:**
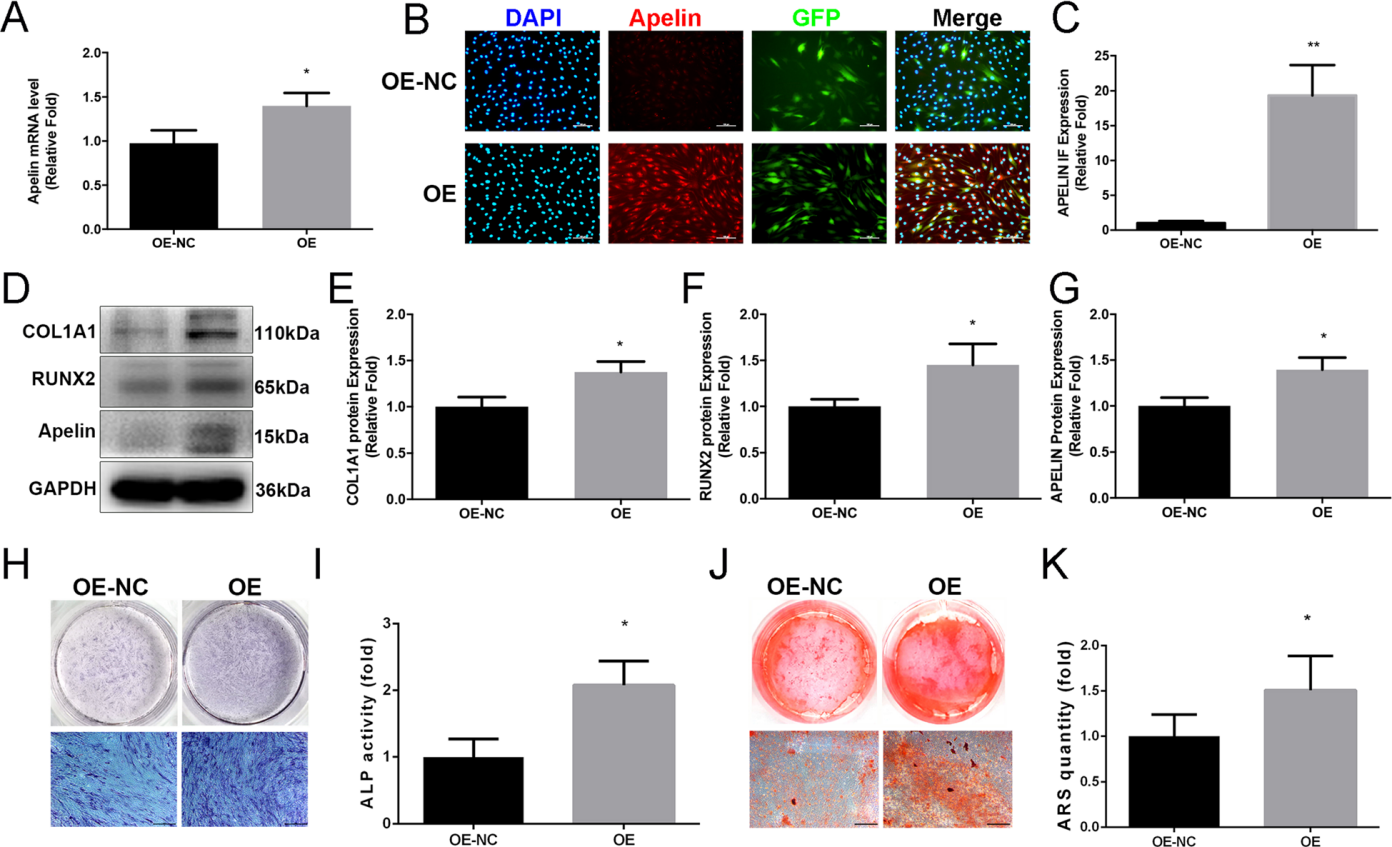
Establishment of Apelin overexpression hBMSC cell line and effects of Apelin on osteogenic differentiation of hBMSCs. **a**–**d** Verification of Apelin overexpression in hBMSCs. Scale bars, 100 μm. **e**–**g** Relative expression of osteo-specific proteins (RUNX2 and COL1A1) was significantly upregulated on day 3 of osteogenesis. The mRNA expression levels were normalized to that of 18S ribosomal RNA. **h**, **i** Staining detection of ALP activity on day 3 of osteogenesis. Scale bars, 500 μm. **j**, **k** Alizarin Red S staining on day 10. Alizarin Red S-stained area was determined by measuring the absorbance at 560 nm. Scale bars, 500 μm. Data are expressed as mean ± SD. Reactions were performed in triplicate. *P < 0.05 and **P < 0.01 compared with the control group

To clarify the roles of endogenous Apelin during osteogenic differentiation of hBMSCs, we constructed an Apelin overexpression hBMSC cell line through lentiviral vectors. Three days after infection and screening, Apelin expression level was determined by qPCR and Western blot analyses. In comparison with the control group, mRNA and protein levels of Apelin were significantly upregulated. Apelin protein expression was also assessed by immunofluorescence (IF) assay, and substantially higher Apelin expression level was observed in the Apelin overexpression group relative to the control group (Fig. 4a–d).

Western blot analysis showed higher expression levels of COL1A1 and RUNX2 proteins in the OE (APELIN overexpression) group compared with the OE-NC (APELIN overexpression control) group on day 3 of osteogenic differentiation (Fig. 4e–g). Compared with the OE-NC group, the activity of ALP increased in the OE group on day 3 of osteogenic differentiation (Fig. 4h, i). In addition, calcium mineralization was markedly enhanced in the OE group relative to the OE-NC group on day 9 (Fig. 4j, k).

### Addition of exogenous recombinant Apelin-13 accelerated bone healing in a rat tibial osteotomy model

**Fig. 5:**
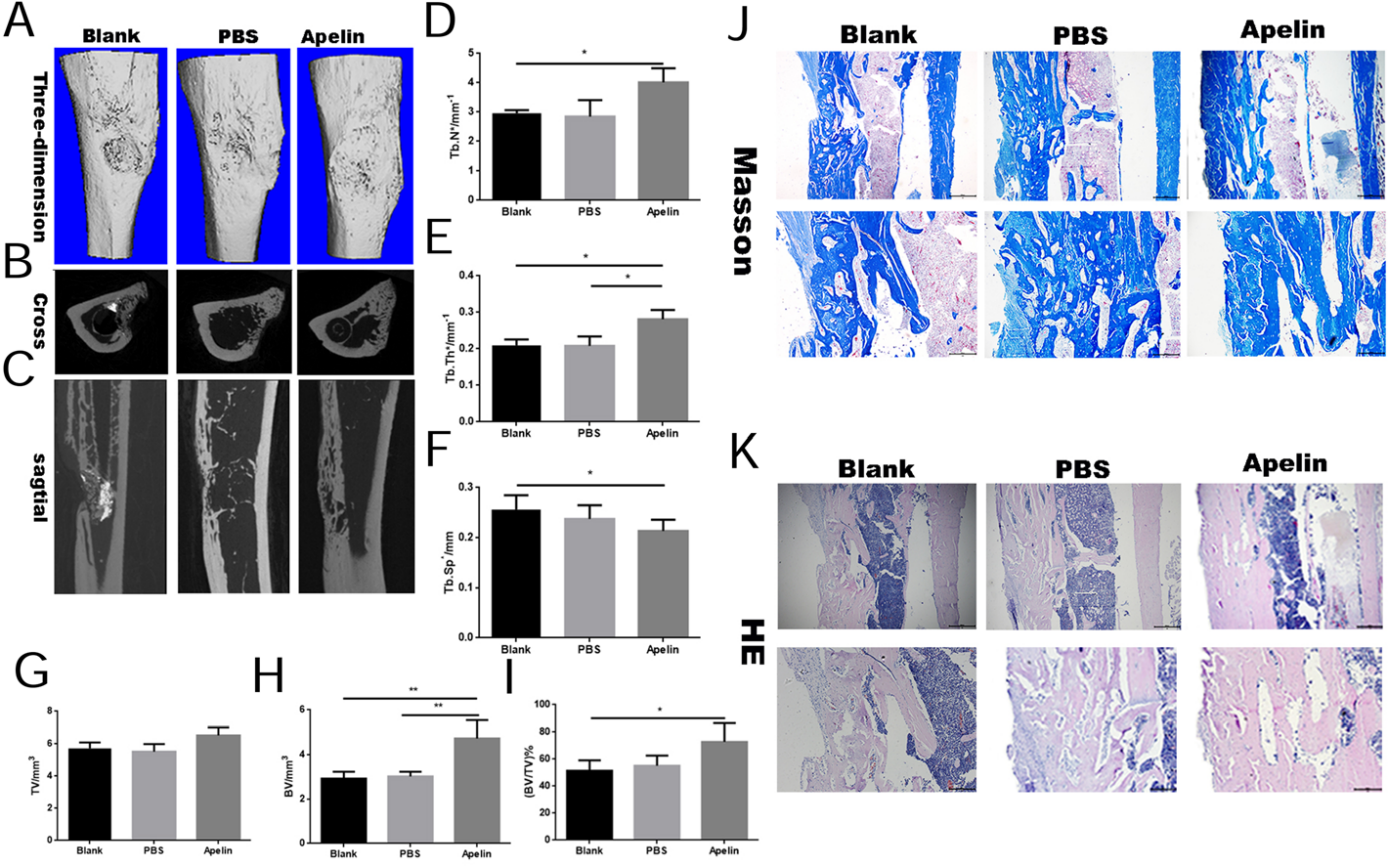
Exogenous recombinant Apelin-13 accelerated bone healing in a rat tibial osteotomy model. **a**–**c** Microcomputed tomography analysis for bone healing. **d**–**i** Bone volume and trabecular thickness were analyzed by microcomputed tomography. Data are expressed as mean ± SD. Assays were performed in triplicate. *P < 0.05 compared with the blank group. **j**, **k** Histological analysis for bone healing. HE, hematoxylin and eosin staining; Masson, Masson’s trichrome staining. Scale bars, 500 μm

To prove the effects of Apelin-13 in vivo, exogenous Apelin-13 was administered in a rat tibial defect model. Microcomputed tomography (μCT) analysis revealed that Apelin-13 promoted fracture healing (Fig. 5a–i). At 6 weeks of age, Apelin-13 significantly reduced the gap distance of cortical defect in comparison with the other two groups (Fig. 5a–c). The trabecular number and trabecular thickness were increased by Apelin-13 treatment (Fig. 5d, e). In addition to the trabecular phenotype, Apelin-13 also increased the thickness of the cortical bone (Fig. 5f–i). Histological analysis demonstrated better cortex growth in the Apelin group compared with the other groups. (Fig. 5j, k).

## Discussion

Successful osteogenesis of MSCs is essential for bone healing [26, 27]. Herein, we provide the first evidence that Apelin may serve as a novel target for promoting the osteogenesis of hBMSCs.

Endogenous Apelin is constantly expressed in the osteogenesis process of hBMSCs, and previous studies have indicated that Apelin plays pivotal roles in bone metabolism. In accordance with our results, Apelin has been shown to suppress apoptosis in both BMSCs and the osteoblastic cell line MC3T3-E1, while exerts no effect on BMSC proliferation [20, 28]. In hemodialysis (HD) patients with secondary hyperparathyroidism, higher PTH levels group had higher apelin levels [1.17 (0.7) ng/ml] compared with lower PTH levels group [0.50 (0.15) ng/ml], and Apelin protects human osteoblasts against apoptosis and induce osteoblast proliferation through competing with the opposite function of PTH [29]. Apelin deficiency has been shown to contribute to the severe metabolic disturbance of the skeletal system [30, 31]. Our results revealed that Apelin-13 significantly upregulated the expression of osteogenic specific genes and proteins in hBMSCs. Meanwhile, ALP activity and mineral deposition were also enhanced by exogenous rhApelin-13 protein or overexpression of Apelin.

In Apelin-13-deficient mice, decreased expression of Wnt/β-catenin signaling pathway-related molecules and downregulated collagen maturation-associated genes (loxl3 and loxl4) was observed [31]. Wnt comprises a large family of secreted glycoproteins that control cellular proliferation, differentiation, migration, apoptosis, survival, and polarity in various cell types [32]. Wnt proteins are known to play critical roles during skeletal patterning [33]. The Wnt receptors include 10 Frizzled family members, ROR2, Ryk, low-density lipoprotein receptor-related protein 5 (LRP5) and LRP6. Different Wnt proteins recognize their cognate receptors and activate at least three different intracellular signaling cascades: the canonical Wnt pathway (also known as the Wnt/β-catenin pathway), the non-canonical Wnt pathway, and the Wnt-calcium pathway. Wnt proteins activate the Frizzled/LRP5 or Frizzle/LRP6 receptor complexes and thus stabilize β-catenin in the cytoplasm. Subsequently, β-catenin is capable of entering the nucleus and regulaing the expression of Wnt target genes [34]. The canonical Wnt/β-catenin signaling pathway has been shown to be closely associated with osteoblastogenesis [35–37] and to be one of the key regulators of skeletal lineage differentiation [38, 39]. In our study, both the total β-catenin and active β-catenin expression levels increased dramatically in response to Apelin-13 during osteogenic differentiation. Moreover, the β-catenin-dependent Wnt signaling pathway inhibitor DKK1 almost completely abrogated the effects of Apelin-13 on osteogenesis. The results indicate that Apelin-13 promotes osteogenic differentiation of hBMSCs mainly through the Wnt/β-catenin signaling pathway.

Lysyl oxidase genes (lox) including loxl3 and loxl4 are collagen maturation-associated genes, which are crucial to cross-link with collagen molecules through enzyme process [40]. What is the link between Apelin and lox genes expression needed further investigation.

Previous studies have demonstrated that growth factors, such as bone morphogenetic proteins (BMPs), promote bone defect healing in vivo [41–45]. In our study, recombinant human Apelin-13 was shown to accelerate bone healing in a rat tibial osteotomy model.

However, our study has some limitations. First, although exogenous Apelin regulates the osteogenic differentiation of hBMSCs by activating Wnt/β-catenin signaling pathway, we do not identify relevant cell surface receptor which mediates the transmission of signals. How Apelin/APJ system interacted with osteogenic signaling pathway needed a further study. Second, it remains unclear that whether accelerated osteogenic effect by endogenous overexpression Apelin through the same way as exogenous Apelin proteins. As mentioned above, the process of bone healing includes endochondral ossification and intramembranous ossification [27]. In this study, we have just investigated intramembranous ossification partially; however, the effects of the Apelin protein on the processes of endochondral ossification remains unclear. From the histological analysis, we can see better cortex growth treated with Apelin. However, a more quantitative analysis is needed to verify the new bone formation by Apelin. And because the small bone defect model used in our vivo study can heal spontaneously, the effect of Apelin is needed to be verified for larger bone defect model. At last, previous study has revealed that in normal healthy human subjects, the concentration of Apelin in plasma was 3.58 ± 0.33 ng/ml [46]; unfortunately, it is unknown how plasma Apelin changed in bone fracture patients. Thus, we think further studies are needed.

## Conclusions

Taken together, these findings indicate that Apelin regulates osteogenic differentiation of hMSCs partly via the Wnt/β-catenin signaling pathway and effectively promotes fracture healing.

## Additional files

Additional file 1: ** Figure S2A.** The expression of ALP mRNA increased significantly on days 1 and 3. Data are expressed as mean ± SD. Assays were performed in triplicate. *P < 0.05 compared with the control group. (TIF 46 kb)

Additional file 2: ** Figure S2B. **Immunofluorescence staining of RUNX2 and COL1A1 proteins on day 3 of osteogenic differentiation (red). Cell nuclei were counter-stained with DAPI (blue). Scale bars, 100 μm. Data are expressed as mean ± SD. Assays were performed in triplicates. *P < 0.05, **P < 0.01 compared with the control group. (TIF 1198 kb)

## Data Availability

The datasets used and/or analyzed during the current study are available from the corresponding author on reasonable request.

## Abbreviations

ALP: Alkaline phosphatase
ARS: Alizarin Red staining
CCK-8: Cell Counting Kit-8
COL1A1: Alpha-1 type I collagen
ddH_2_O: Double distilled water
EDTA: Ethylene diaminetetra acetic acid
GAPDH: Glyceraldehyde-3-phosphate dehydrogenase
hBMSCs: Human bone marrow-derived mesenchymal stem cells
HE: Hematoxylin and eosin
IF: Immunofluorescence
L-DMEM: Low-sugar Dulbecco’s modified Eagle’s medium
LRP5: Lipoprotein receptor-related protein 5
MAPK: Mitogen-activated protein kinase
OCN: Osteocalcin
OE: Overexpression group
OE-NC: Overexpression control group
PI3K: Phosphoinositide 3-kinase
RUNX2: Runt-related transcription factor 2
