# Supplementary figures and images for: Apelin enhances the osteogenic differentiation of human bone marrow mesenchymal stem cells partly through Wnt/β-catenin signaling pathway
Source: Stem Cell Res Ther. 2019 Jun 25;10:189. doi: 10.1186/s13287-019-1286-x (PMC6593611; doi:10.1186/s13287-019-1286-x)

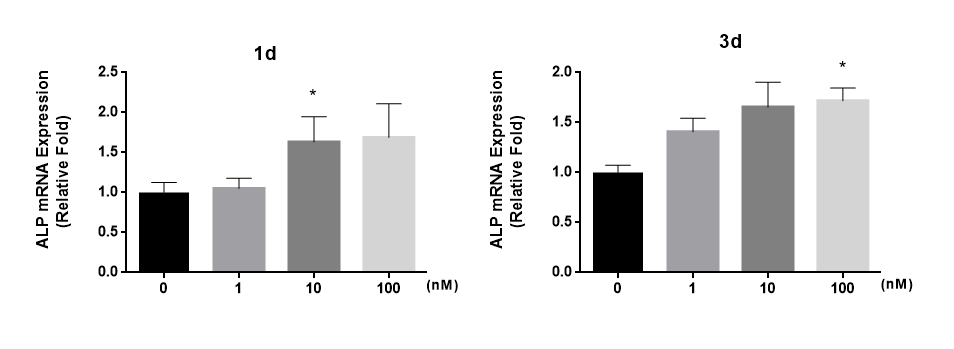

Supplement: Supplementary file 1 — Figure S2A. The expression of ALP mRNA increased significantly on days 1 and 3. Data are expressed as mean ± SD. Assays were performed in triplicate. *P < 0.05 compared with the control group. (TIF 46 kb) [file 13287_2019_1286_MOESM1_ESM.tif]

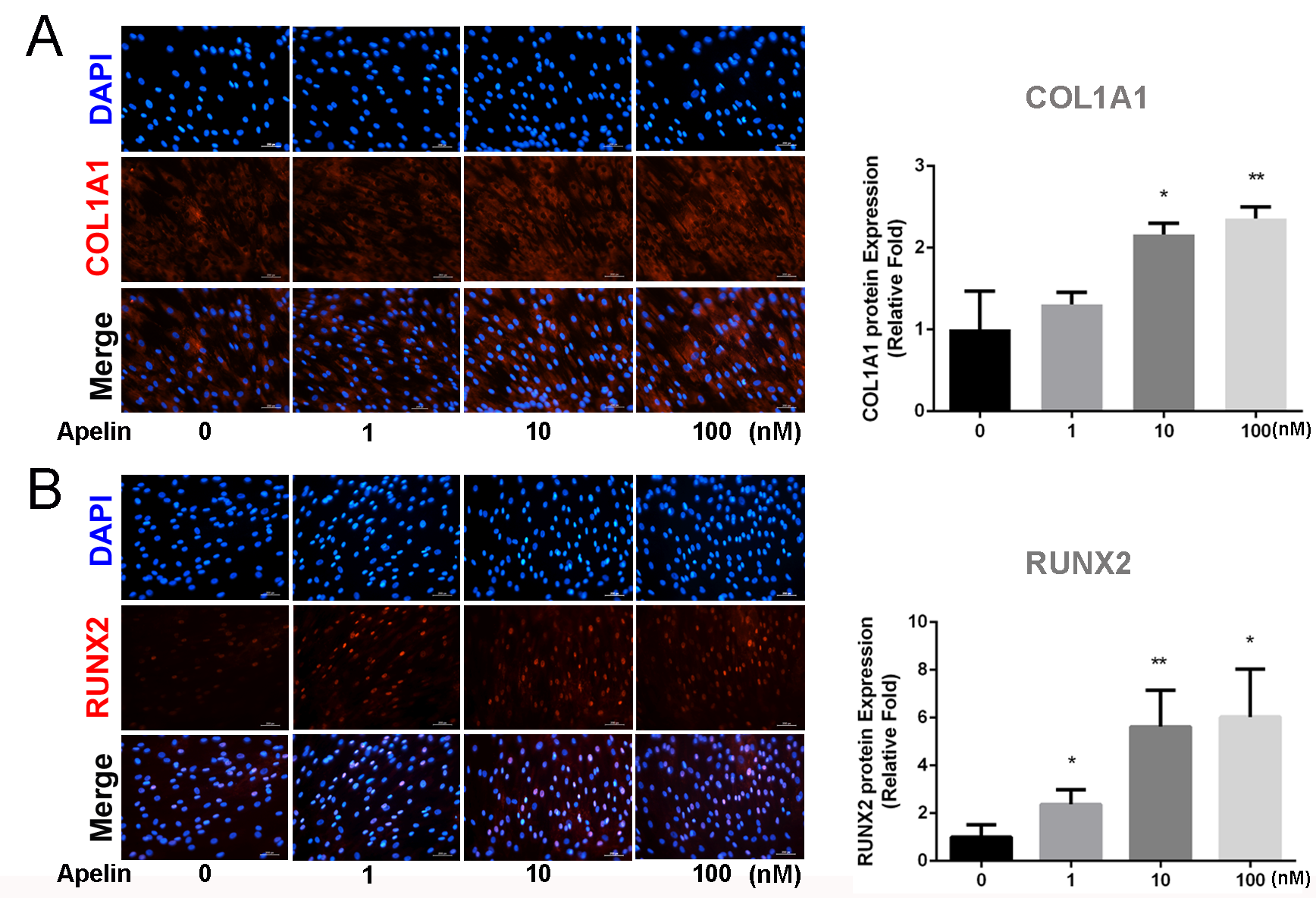

Supplement: Supplementary file 2 — Figure S2B. Immunofluorescence staining of RUNX2 and COL1A1 proteins on day 3 of osteogenic differentiation (red). Cell nuclei were counter-stained with DAPI (blue). Scale bars, 100 μm. Data are expressed as mean ± SD. Assays were performed in triplicates. *P < 0.05, **P < 0.01 compared with the control group. (TIF 1198 kb) [file 13287_2019_1286_MOESM2_ESM.tif]
